# Supplementary material for: Stn1 supports Mec1 function in protecting stalled replication forks from degradation
Source: PLoS Genet. 2025 Oct 15;21(10):e1011917. doi: 10.1371/journal.pgen.1011917 (PMC12548912; doi:10.1371/journal.pgen.1011917)
Supplement: S1 Table — (DOCX) [file pgen.1011917.s001.docx]

**S1 Table. HADDOCK 2.4 binding-energy evaluation for water-refined models of the CST wild-type and CS^L60F^T mutant complexes.**

|  | **Model 1** | **Model 2** | **Model 3** | **Model 4** | **Average** | **dev st** | **t-test** |
| --- | --- | --- | --- | --- | --- | --- | --- |
| **CST** | -765,847 | -529,311 | -680,109 | -722,484 | -674,438 | 102,8883 | 0,021169 |
| **CS^L60F^T** | -846,36 | -846,68 | -881,338 | -940,229 | -878,652 | 44,21132 |  |
