# Supplementary material for: Stn1 supports Mec1 function in protecting stalled replication forks from degradation
Source: PLoS Genet. 2025 Oct 15;21(10):e1011917. doi: 10.1371/journal.pgen.1011917 (PMC12548912; doi:10.1371/journal.pgen.1011917)
Supplement: S2 Table — (DOCX) [file pgen.1011917.s002.docx]

**S2 Table. *S. cerevisiae* strains used in this study.**

| **Strain** | **Relevant genotype** | **Source** |
| --- | --- | --- |
| W303 | *MAT*a/α *ade2-1 can1-100 his3-11,15 leu2-3,112 trp1-1 ura3-1 rad5-535* |  |
| DMP7876/22B | W303 *MAT*a *stn1-L60F::URA3* | This study |
| YLL2804.1 | W303 *MAT*a *stn1-*Δ*C::KANMX* | 1 |
| DMP3307/3C | W303 *MAT*a *mec1-100::LEU2::mec1*Δ | 2 |
| DMP7880/4B | W303 *MAT*a *mec1-100::LEU2::mec1*Δ *stn1-L60F::URA3* | This study |
| DMP8036/5B | W303 *MAT*a *mec1-100::LEU2::mec1*Δ *stn1-*Δ*C::KANMX* | This study |
| YLL2614.7 | W303 *MAT*a *STN1-3HA::URA3* | 3 |
| DMP8026/2C | W303 *MAT*a *stn1-L60F::URA3-3HA::TRP1* | This study |
| DMP8141 | *MAT*a*/α* *mec1-100::LEU2::mec1*Δ*/ mec1-100::LEU2::mec1*Δ *stn1-L60F::URA3 stn1-L60F::URA3* | This study |
| DMP8142 | *MAT*a*/α* *mec1-100::LEU2::mec1*Δ*/ mec1-100::LEU2::mec1*Δ *STN1*/ *stn1-L60F::URA3* | This study |
| DMP8143 | *MAT*a*/α* *mec1-100::LEU2::mec1*Δ*/ mec1-100::LEU2::mec1*Δ *STN1*/ *STN1* | This study |
| DMP8129/1D | W303 *MAT*a *mec1-100::LEU2::mec1*Δ *stn1-L60F::URA3 pol12-216/13XMYC/SpHis5*+ | This study |
| DMP8115/4B | W303 *MAT*a *mec1-100::LEU2::mec1*Δ  *pol12-216/13XMYC/SpHis5*+ | This study |
| DMP8129/2D | W303 *MAT*a *stn1-L60F::URA3 pol12-216/13XMYC/SpHis5*+ | This study |
| YSG406 | W303 *MAT*a *pol12-216/13XMYC/SpHis5*+ | 4 |
| DMP8037/1B | W303 *MAT*a *mec1-100::LEU2::mec1*Δ *stn1-*Δ*C::KANMX exo1*Δ*::LEU2* | This study |
| DMP6509/5B | W303 *mec1-100::LEU2::mec1*Δ *exo1*Δ*::LEU2* | 5 |
| DMP8037/8D | W303 *MAT*a *stn1-*Δ*C::KANMX* *exo1*Δ*::LEU2* | This study |
| DMP6509/1B | W303 *exo1*Δ*::LEU2* | 2 |
| DMP8061/9A | W303 *MAT*a *mec1-100::LEU2::mec1*Δ *stn1-*Δ*C::KANMX mre11-H125N::HPHMX* | This study |
| DMP8036/7A | W303 *MAT*a *mec1-100::LEU2::mec1*Δ  *mre11-H125N::HPHMX* | This study |
| DMP8036/4C | W303 *MAT*a *stn1-*Δ*C::KANMX mre11-H125N::HPHMX* | This study |
| YLL4478.1 | W303 *MAT*a *mre11-H125N::HPHMX* | This study |
| DMP8051/9A | W303 *MAT*a *mec1-100::LEU2::mec1*Δ *stn1-*Δ*C::KANMX sgs1*Δ*::TRP1* | This study |
| DMP8051/8D | W303 *MAT*a *stn1-*Δ*C::KANMX sgs1*Δ*::TRP1* | This study |
| DMP5056/7C | W303 *MAT*a *sgs1*Δ*::TRP1* | 6 |
| DMP8028/4D | W303 *MAT*a *mec1-100::LEU2::mec1*Δ *stn1-L60F::URA3 mre11-H125N::HPHMX* | This study |
| DMP8052/3D | W303 *MAT*α *stn1-L60F::URA3 mre11-H125N::HPHMX* | This study |
| DMP8027/7A | W303 *MAT*a *mec1-100::LEU2::mec1*Δ *stn1-L60F::URA3 exo1*Δ*::HIS3* | This study |
| DMP8037/2D | W303 *MAT*a *mec1-100::LEU2::mec1*Δ *exo1*Δ*::LEU2* | This study |
| DMP7917/6C | W303 *stn1-L60F::URA3 exo1*Δ*::LEU2* | This study |
| DMP8030/6A | W303 *MAT*a *mec1-100::LEU2::mec1*Δ *stn1-L60F::URA3 sgs1*Δ*::TRP1* | This study |
| DMP8051/2B | W303 *MAT*a *mec1-100::LEU2::mec1*Δ *sgs1*Δ*::TRP1* | This study |
| DMP7882/4D | W303 *MAT*a *stn1-L60F::URA3* *sgs1*Δ*::TRP1* | This study |
| DMP8094/33D | W303 *MAT*a *mec1-100::LEU2::mec1*Δ *EXO1-18MYC::HIS3* | This study |
| DMP8064/2C | W303 *MAT*a *mec1-100::LEU2::mec1*Δ *MRE11-18MYC::TRP1* | This study |
| DMP8065/8D | W303 *MAT*a *mec1-100::LEU2::mec1*Δ *SGS1-3HA::URA3* | This study |
| DMP8094/13D | W303 *MAT*a *stn1-*Δ*C::KANMX* *EXO1-18MYC::HIS3* | This study |
| DMP8064/1D | W303 *MAT*a *stn1-*Δ*C::KANMX* *MRE11-18MYC::TRP1* | This study |
| DMP8065/7C | W303 *MAT*a *stn1-*Δ*C::KANMX* *SGS1-3HA::URA3* | This study |
| DMP8119/1D | W303 *MAT*a *stn1-L60F::URA3* *EXO1-18MYC::HIS3* | This study |
| DMP8120/6C | W303 *MAT*a *stn1-L60F::URA3* *MRE11-18MYC::TRP1* | This study |
| DMP8121/1A | W303 *MAT*a *stn1-L60F::URA3 SGS1-3HA::URA3* | This study |
| DMP8094/28D | W303 *MAT*a *mec1-100::LEU2::mec1*Δ *stn1-*Δ*C::KANMX* *EXO1-18MYC::HIS3* | This study |
| DMP8064/12B | W303 *MAT*a *mec1-100::LEU2::mec1*Δ *stn1-*Δ*C::KANMX* *MRE11-18MYC::TRP1* | This study |
| DMP8065/1C | W303 *MAT*a *mec1-100::LEU2::mec1*Δ *stn1-*Δ*C::KANMX* *SGS1-3HA::URA3* | This study |
| DMP8119/3B | W303 *MAT*a *mec1-100::LEU2::mec1*Δ *stn1-L60F::URA3* *EXO1-18MYC::HIS3* | This study |
| DMP8121/2C | W303 *MAT*a *mec1-100::LEU2::mec1*Δ *stn1-L60F::URA3 SGS1-3HA::URA3* | This study |
| DMP8122/9B | W303 *MAT*a *mec1-100::LEU2::mec1*Δ *stn1-L60F::URA3 MRE11-18MYC::TRP1* | This study |
| JKM139 | *MAT*a *hml*Δ*::ADE1*, *hmr*Δ*::ADE1*, *ade1-100, lys5,*  *leu2-3,112, trp1::hisG ura3-52, ho, ade3::GAL-HO site* | 7 |
| DMP7936/3C | JKM139 *MAT*a *stn1-L60F::URA3* | This study |
| DMP7937/2A | JKM139 *MAT*a *stn1-*Δ*C::KANMX* | This study |
| DMP5996/1C | JKM139 *MAT*a *SGS1-3HA::URA3* | 6 |
| DMP8118/7B | JKM139 *MAT*a *SGS1-3HA::URA3* *stn1-L60F::URA3* | This study |
| DMP8072/3B | JKM139 *MAT*a *SGS1-3HA::URA3* *stn1-*Δ*C::KANMX* | This study |
| YLL1959.2 | JKM139 *MAT*a *EXO1-18MYC::TRP1* | 5 |
| DMP8117/1B | JKM139 *MAT*a *EXO1-18MYC::TRP1* *stn1-L60F::URA3* | This study |
| DMP8073/1D | JKM139 *MAT*a *EXO1-18MYC::TRP1* *stn1-*Δ*C::KANMX* | This study |
| YLL1854.2 | JKM139 *MAT*a *MRE11-18MYC::TRP1* | 8 |
| DMP8116/2D | JKM139 *MAT*a *MRE11-18MYC::TRP1* *stn1-L60F::URA3* | This study |
| DMP8071/2B | JKM139 *MAT*a *MRE11-18MYC::TRP1* *stn1-*Δ*C::KANMX* | This study |
| DMP8177/3C | W303 *MAT*a *STN1-3HA::URA3* *ku70Δ::KANMX* | This study |
| DMP8186/4D | W303 *MAT*a *stn1-L60F::URA3-3HA::TRP1* *ku70Δ::KANMX* | This study |
| DMP8181/1B | W303 *MAT*a *pol1-236::HPHMX pol12-216/13XMYC/SpHis5*+ *stn1-L60F::URA3* | This study |
| DMP8182/3C | W303 *MAT*a *pol1-236::HPHMX pol12-216/13XMYC/SpHis5*+ | This study |
| DMP8187/17D | W303 *MAT*a *mec1-100::LEU2::mec1*Δ *pol1-236::HPHMX pol12-216/13XMYC/SpHis5*+ *stn1-L60F::URA3* | This study |
| DMP8188/4B | W303 *MAT*a *mec1-100::LEU2::mec1*Δ *pol1-236::HPHMX pol12-216/13XMYC/SpHis5*+ | This study |
| DMP7881/2C | W303 *MAT*a *mec1Δ::HIS3 sml1Δ::KANMX stn1-L60F::URA3* | This study |
| YLL490.4 | W303 *MAT*a *mec1Δ::HIS3 sml1Δ::KANMX* | 9 |

**References**

1. Anbalagan S, Bonetti D, Lucchini G, Longhese MP. Rif1 supports the function of the CST complex in yeast telomere capping. PLoS Genet. 2011;7: e1002024. doi:10.1371/journal.pgen.1002024
2. Villa M, Bonetti D, Carraro M, Longhese MP. Rad9/53BP1 protects stalled replication forks from degradation in Mec1/ATR-defective cells. EMBO Rep. 2018;19: 351-367. doi:10.15252/embr.201744910
3. Cesena D, Cassani C, Rizzo E, Lisby M, Bonetti D, Longhese MP. Regulation of telomere metabolism by the RNA processing protein Xrn1. Nucleic Acids Res. 2017;45: 3860-3874. doi:10.1093/nar/gkx072
4. Grossi S, Puglisi A, Dmitriev PV, Lopes M, Shore D. Pol12, the B subunit of DNA polymerase alpha, functions in both telomere capping and length regulation. Genes Dev. 2004;18: 992-1006. doi:10.1101/gad.300004
5. Manfrini N, Trovesi C, Wery M, Martina M, Cesena D, Descrimes M, Morillon A, d'Adda di Fagagna F, Longhese MP. RNA-processing proteins regulate Mec1/ATR activation by promoting generation of RPA-coated ssDNA. EMBO Rep. 2015;16: 221-231. doi: 10.15252/embr.201439458.
6. Gnugnoli M, Rinaldi C, Casari E, Pizzul P, Bonetti D, Longhese MP. Proteasome-mediated degradation of long-range nucleases negatively regulates resection of DNA double-strand breaks. iScience. 2024;27: 110373. doi:10.1016/j.isci.2024.110373
7. Lee SE, Moore JK, Holmes A, Umezu K, Kolodner RD, Haber JE. *Saccharomyces* Ku70, Mre11/Rad50 and RPA proteins regulate adaptation to G2/M arrest after DNA damage. Cell. 1998;94: 399-409. doi: 10.1016/s0092-8674(00)81482-8.
8. Cassani C, Gobbini E, Wang W, Niu H, Clerici M, Sung P, Longhese MP. Tel1 and Rif2 regulate MRX functions in end-tethering and repair of DNA double-strand breaks. PLoS Biol. 2016;14: e1002387. doi: 10.1371/journal.pbio.1002387.
9. Longhese MP, Paciotti V, Neecke H, Lucchini G. Checkpoint proteins influence telomeric silencing and length maintenance in budding yeast. Genetics. 2000;155: 1577-1591. doi:10.1093/genetics/155.4.1577
