# Supplementary material for: Stn1 supports Mec1 function in protecting stalled replication forks from degradation
Source: PLoS Genet. 2025 Oct 15;21(10):e1011917. doi: 10.1371/journal.pgen.1011917 (PMC12548912; doi:10.1371/journal.pgen.1011917)
Supplement: S3 Table — (DOCX) [file pgen.1011917.s003.docx]

**S3 Table. Oligonucleotides used in this study.**

| **Name** | **Sequence (5’-3’)** | **Experiment** |
| --- | --- | --- |
| ARO - | ACCTACAGGAGGACCCGAAA | ChIP DSB |
| ARO + | TGAGTCGTTACAAGGTGATGCC | ChIP DSB |
| MAT 0.2 - | CCCGTATAGCCAATTCGTTC | ChIP DSB |
| MAT 0.2 + | TCAGACTCAAGCAAACAATCAA | ChIP DSB |
| MAT 0.6 - | CATGCGGTTCACATGACTTT | ChIP DSB |
| MAT 0.6 + | CACCCAAGAAGGCGAATAAG | ChIP DSB |
| MAT 1.8 - | CGCGAGTCTTATGCCAAAAA | ChIP DSB |
| MAT 1.8 + | ACGTCGTTGTTAATGGTGGTG | ChIP DSB |
| ARS305 - | TGAAACTGGACATATTTGAGGAATTT | ChIP ARS |
| ARS305 + | TTTGGAGCTCAAGTGGATTGAG | ChIP ARS |
| ARS 607 - | TAATGCACGAGCCGAAACAA | ChIP ARS |
| ARS 607 + | CTTTAGCTGGGTTTATGGGAGG | ChIP ARS |
| ARS607 - 14kb (CNTR) - | GCATGACAGCCGAATCGAT | ChIP ARS |
| ARS607 - 14kb (CNTR) + | CAGGATATGCGGCCAAATTT | ChIP ARS |
| KCC4 - | CTCTGGAAATTTCGGTGTCATTG | DSB resection |
| KCC4 + | TCGTATCAGGTCTGCCCTATGAA | DSB resection |
| R0.15 - | GAGCAAGACGATGGGGAGTTTC | DSB resection |
| R0.15 + | CCTGGTTTTGGTTTTGTAGAGTGG | DSB resection |
| R0.9 - | CGGCATATTTGTATTAACCC | DSB resection |
| R0.9 + | CGATATTAAGTCCTCCGT | DSB resection |
| R1.7 - | TTGCTTCACCAATTTTGGAC | DSB resection |
| R1.7 + | TTTGTTTTGCCTAGAAGACTC | DSB resection |
| R8.9- | GAGTATACCTCTATATGCTGTG | DSB resection |
| R8.9+ | AGTGATGACGTACGTGTATAG | DSB resection |
| R15.8 - | CCATTTGGTAGTATAGCAGC | DSB resection |
| R15.8 + | CCTTGAATGATAGCCTCTTC | DSB resection |
| HO CUT - | TCACCACGTACTTCAGCATA | cutting efficiency |
| HO CUT + | GTGGCATTACTCCACTTCAA | cutting efficiency |
| ARS607 - | CTACTGTGCCGAATAATGTG | ssDNA ARS |
| ARS607 + | ACTAGATCTGGAGTGACCAA | ssDNA ARS |
| ARS607 + 0.5 kb - | ATAGGAGTAACTACGGGGTTA | ssDNA ARS |
| ARS607 + 0.5 kb + | CAACCTACAAGAGGAAACATG | ssDNA ARS |
| ARS607 - 1.7 kb - | ATGACTCGTTCAAGGGATC | ssDNA ARS |
| ARS607 - 1.7 kb + | TTGGGCTCAATCAAAGTCGT | ssDNA ARS |
| ARS607 - 3.0 kb - | TCCGTGATCAATCCATCAAG | ssDNA ARS |
| ARS607 - 3.0 kb + | CTCGGATGGATACCTATACA | ssDNA ARS |
| TRP3 - | ATCATCAGATGACCTTCCTC | ssDNA ARS |
| TRP3 + | CATCCTGCTTGAAGGTTACT | ssDNA ARS |
| CONTROL - | TTACCGTCCGCATTTTGAA | ssDNA ARS |
| CONTROL + | CCACAGGTTTAGATAAGGA | ssDNA ARS |
