# Supplementary figures and images for: Stn1 supports Mec1 function in protecting stalled replication forks from degradation
Source: PLoS Genet. 2025 Oct 15;21(10):e1011917. doi: 10.1371/journal.pgen.1011917 (PMC12548912; doi:10.1371/journal.pgen.1011917)

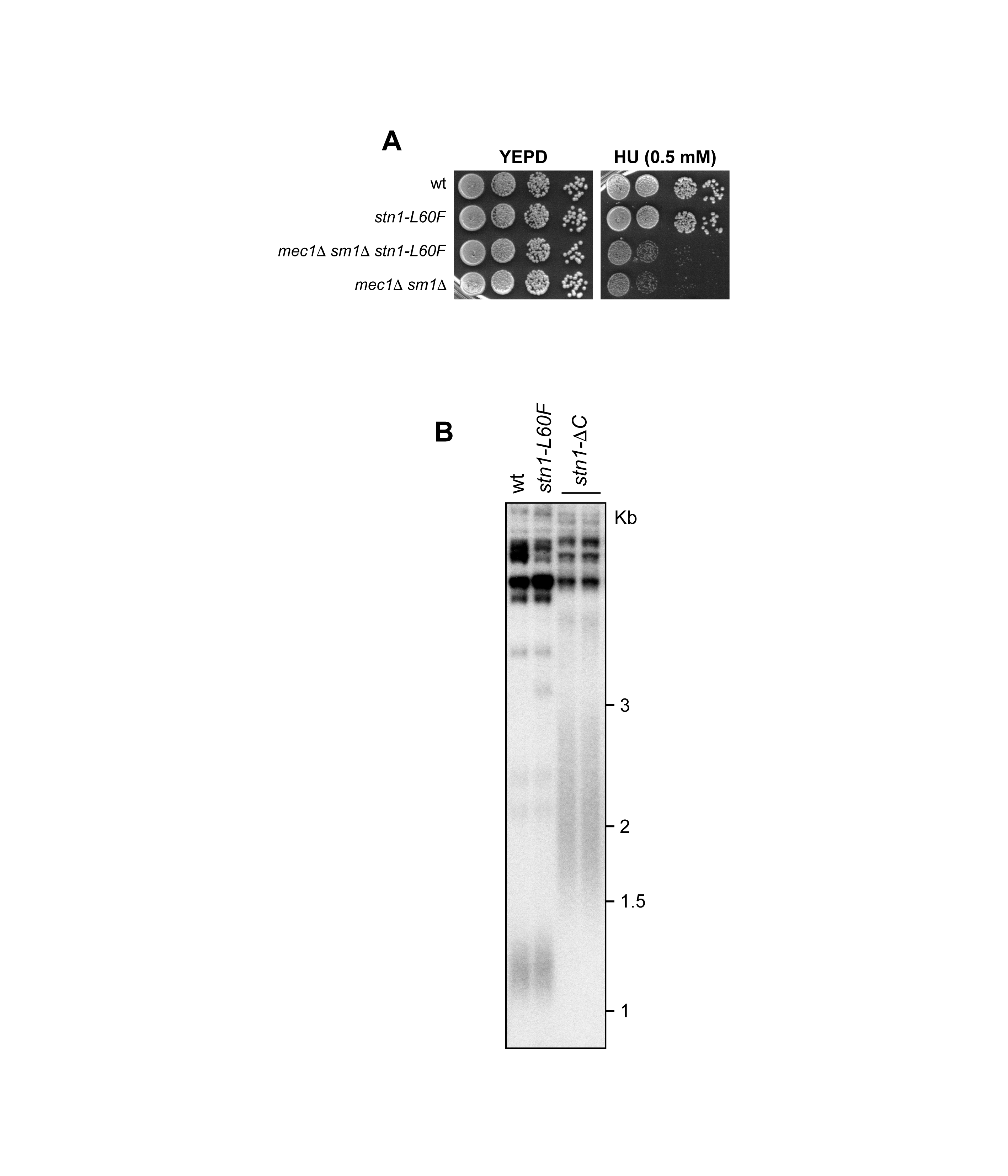

Supplement: S1 Fig — (A) Exponentially growing cell cultures were serially diluted (1:10) and each dilution was spotted out onto YEPD plates with or without HU. (B) XhoI-cut genomic DNA from exponentially growing cells was subjected to Southern blot analysis using a radiolabeled poly(GT) telomere-specific probe. (TIF) [file pgen.1011917.s004.tif]

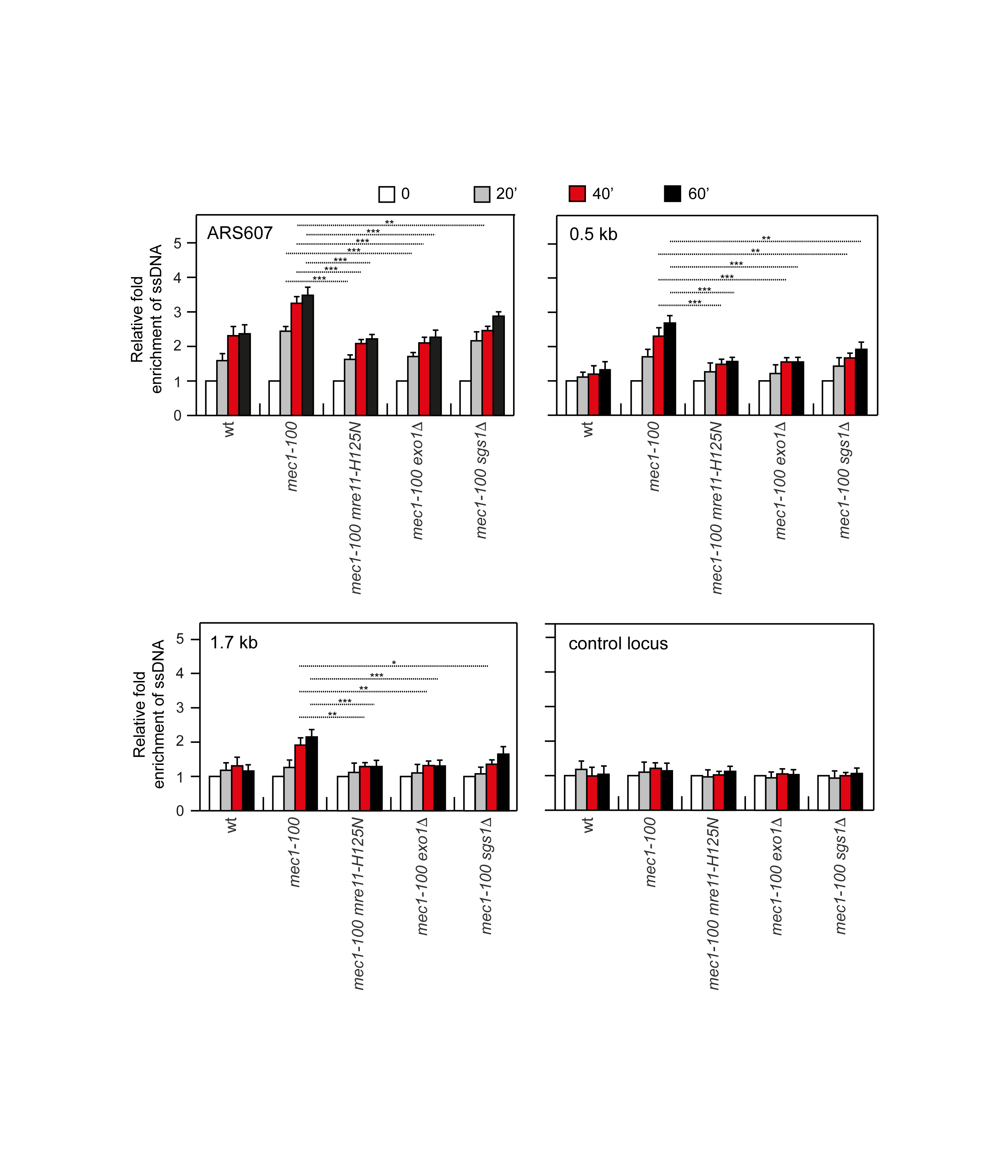

Supplement: S2 Fig — Exponentially growing YEPD cell cultures were arrested in G1 with α-factor (time zero) and then released into YEPD containing 0.2M HU. Genomic DNA prepared at different time points after α-factor release was either digested or mock-digested with SspI and used as a template in qPCR. The value of SspI-digested over non-digested DNAs was determined for each time point after normalization to an amplicon on chromosome XI that does not contain SspI sites. The data shown are expressed as fold-enrichment in ssDNA at different time points after α-factor release in HU relative to the α-factor (time zero) (set to 1.0). A locus containing SspI sites on chromosome XI is used as a control (control locus). Plotted values are the mean values ± s.d. from three independent experiments. ***p < 0.005, **p < 0.01, *p < 0.05 (Student’s t-test). (TIF) [file pgen.1011917.s005.tif]

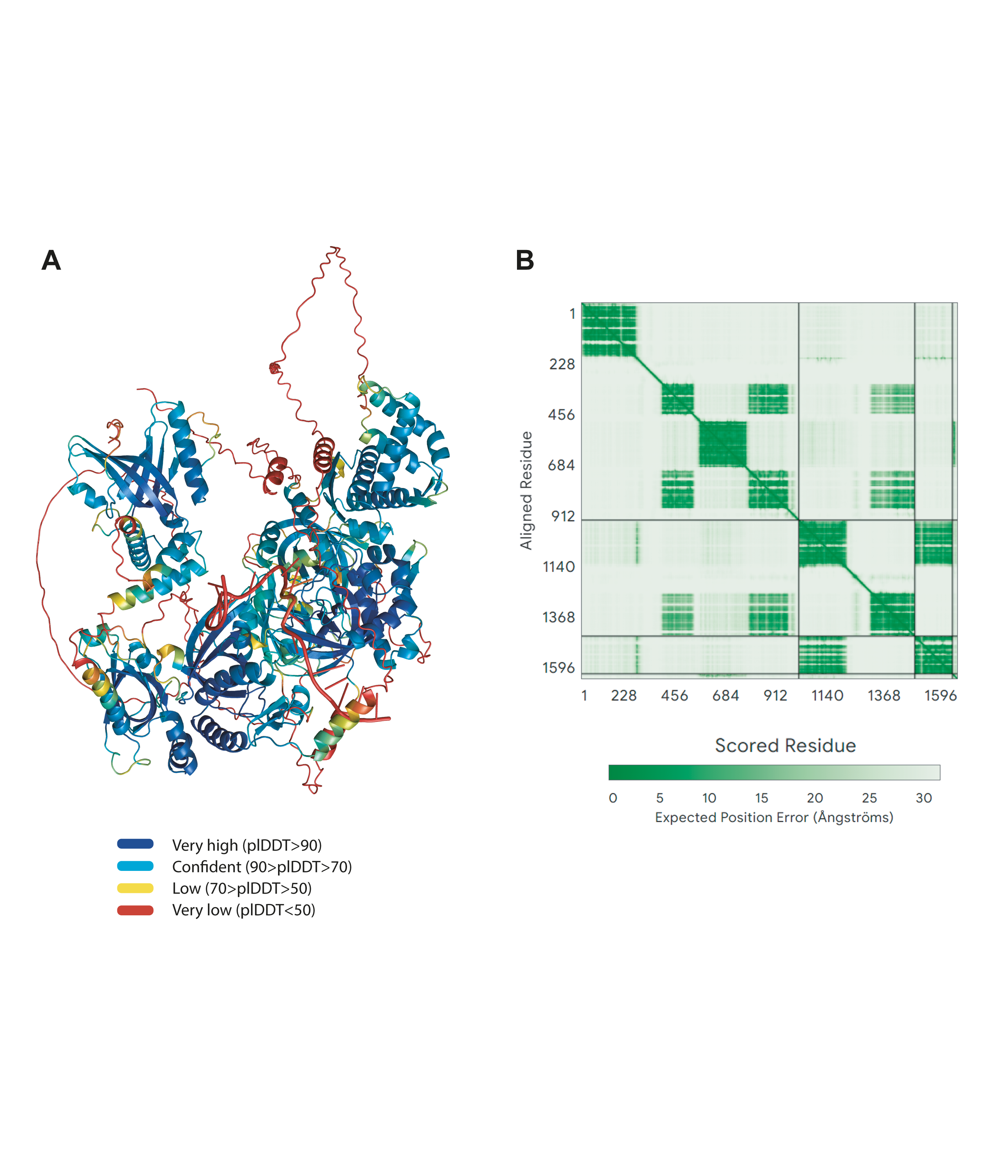

Supplement: S3 Fig — (A) The model generated by AlphaFold 3 for the budding yeast Cdc13-Stn1-Ten1 complex bound to a 20-nt ssDNA is represented as a cartoon and coloured according to the lDDT score. (B) Expected Position Error calculated by AlphaFold 3 predictor is represented for the residues of Cdc13, Stn1, and Ten1, numbered consecutively. (TIF) [file pgen.1011917.s006.tif]

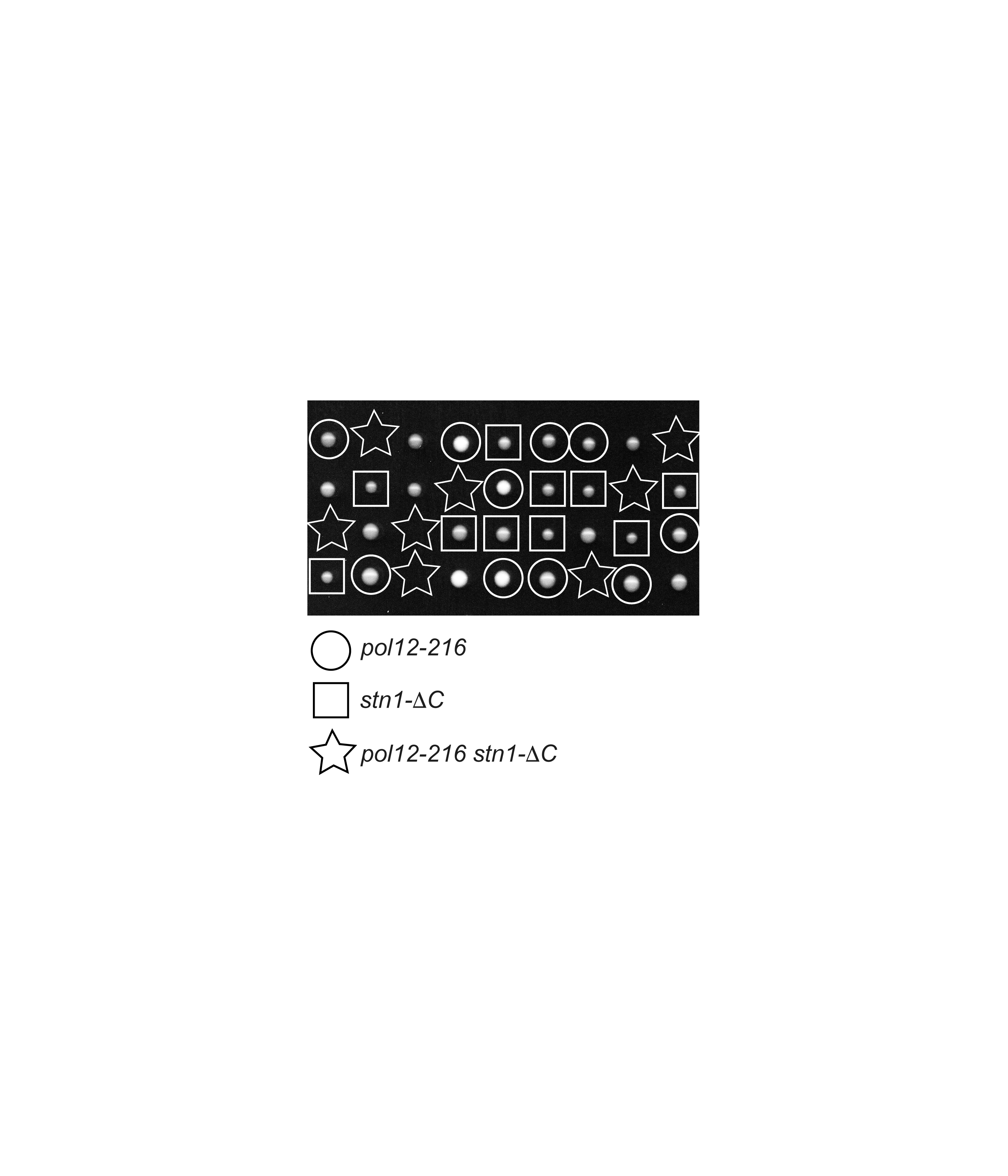

Supplement: S4 Fig — Meiotic tetrads were dissected on YEPD plates that were incubated at 25°C, followed by spore genotyping. (TIF) [file pgen.1011917.s007.tif]

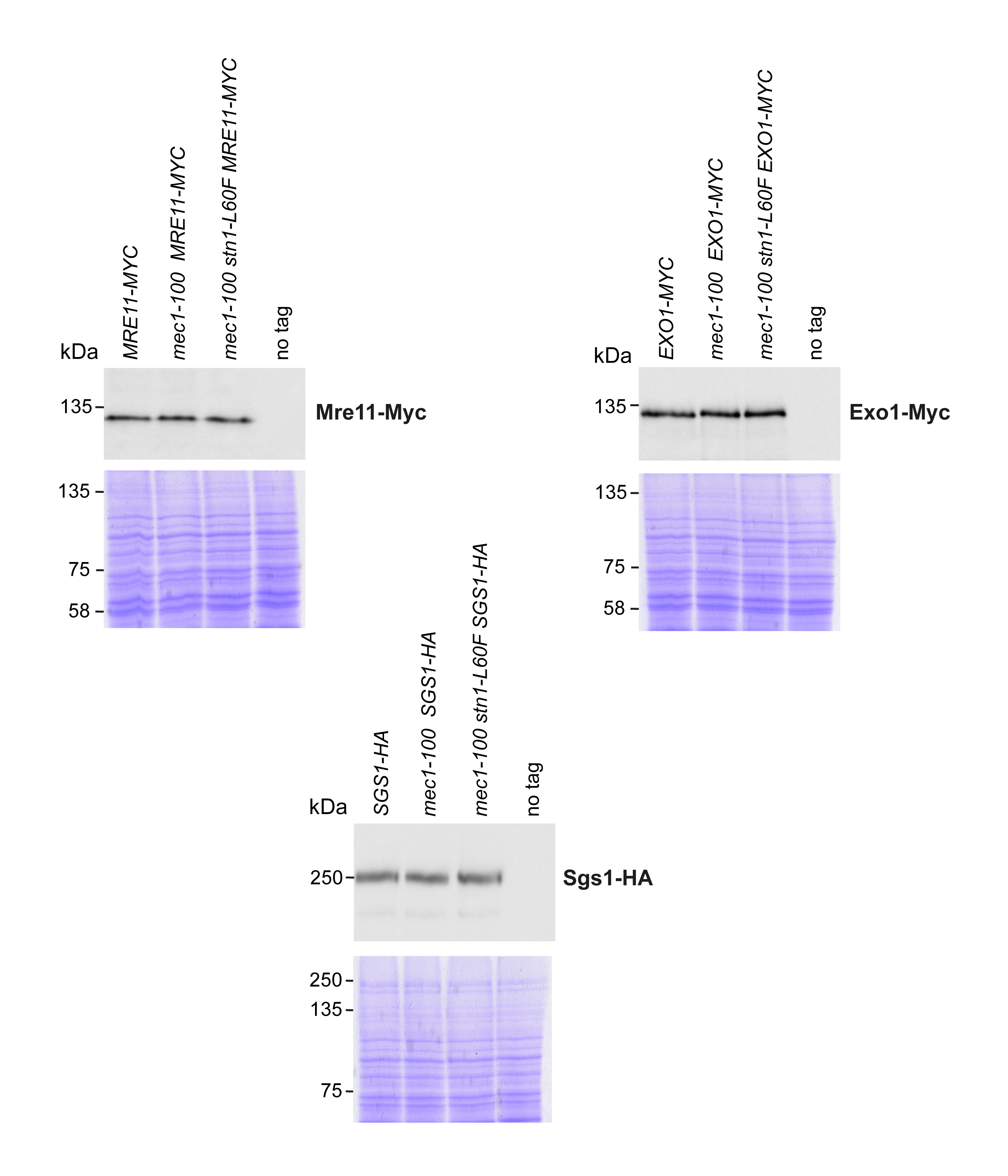

Supplement: S5 Fig — Western blot with an anti-HA or an anti-Myc antibody of extracts used for the ChIP analysis shown in Fig 8. The same amount of extracts was separated by SDS-PAGE and stained with Coomassie Blue as a loading control. (TIF) [file pgen.1011917.s008.tif]

Figure 1E

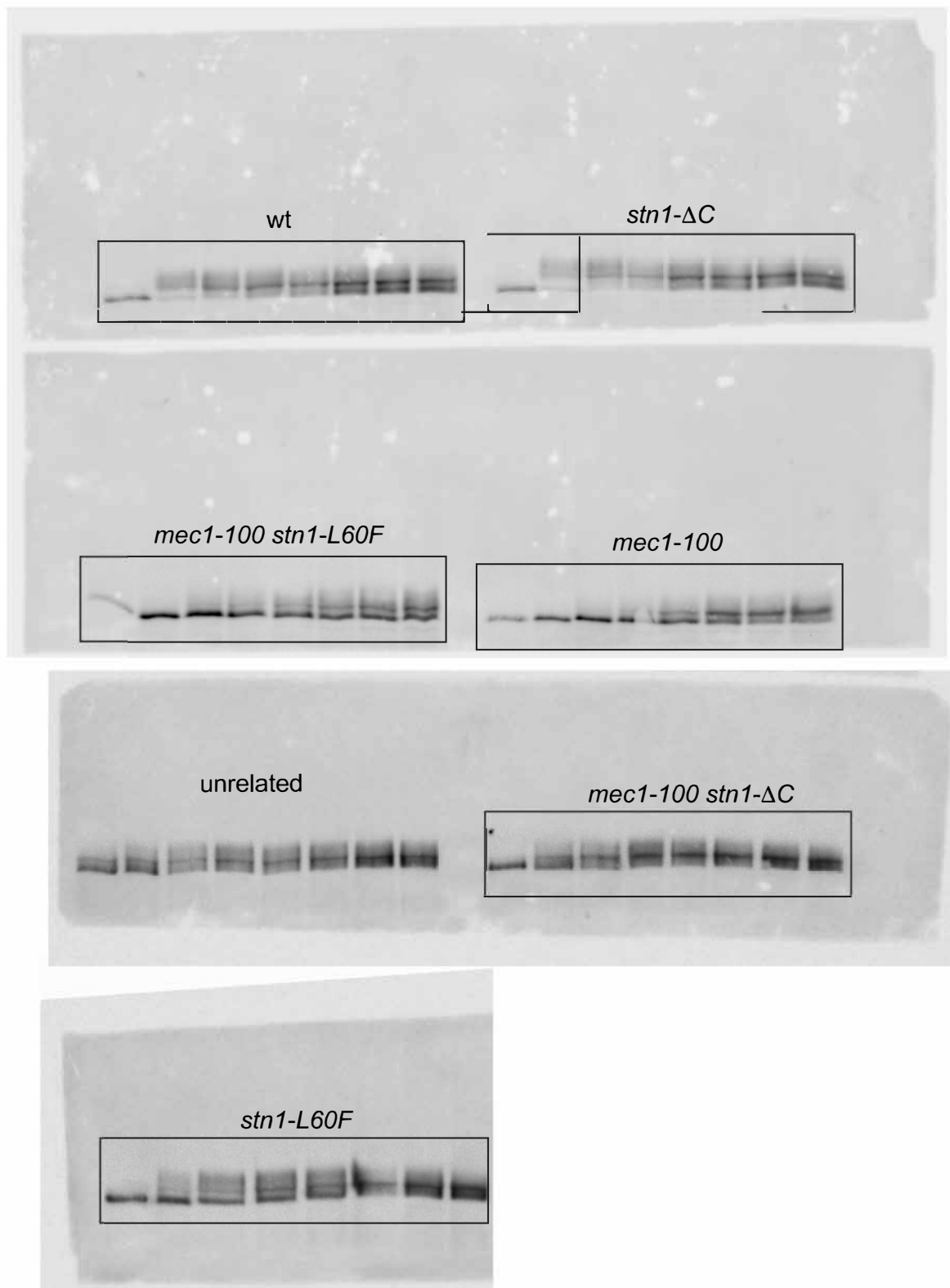

Figure 1G

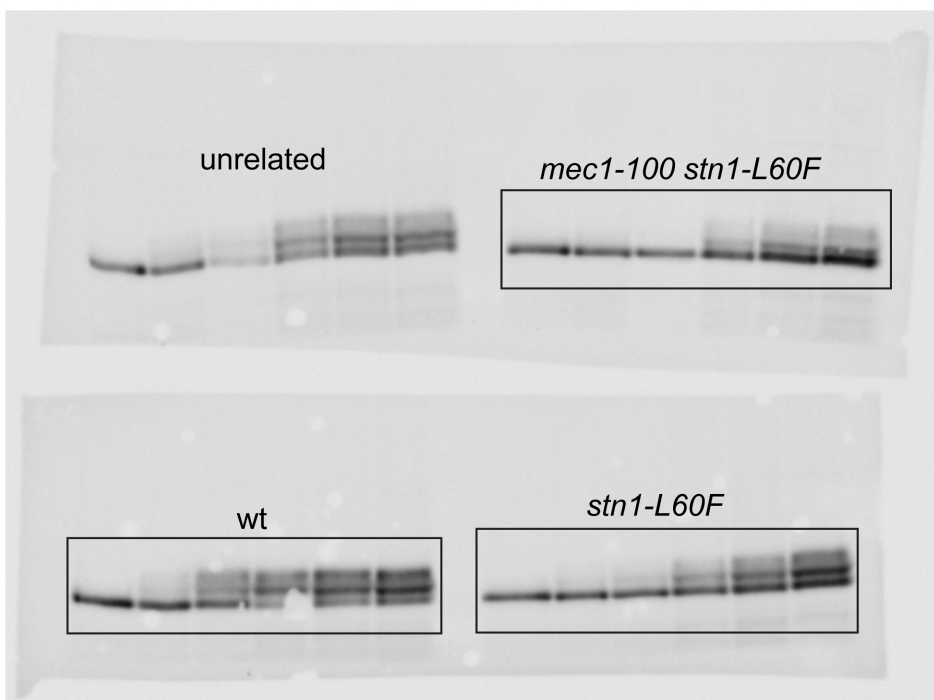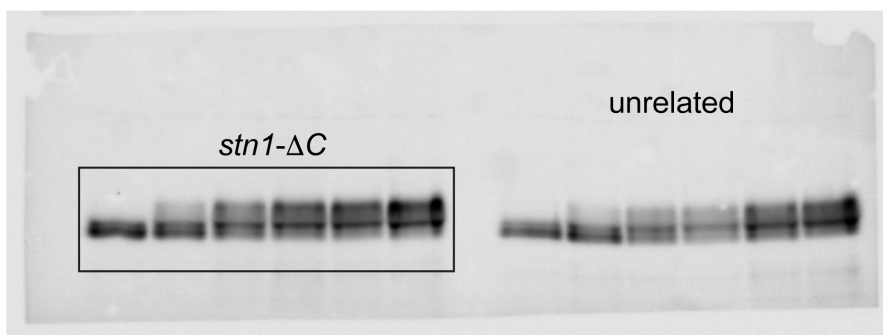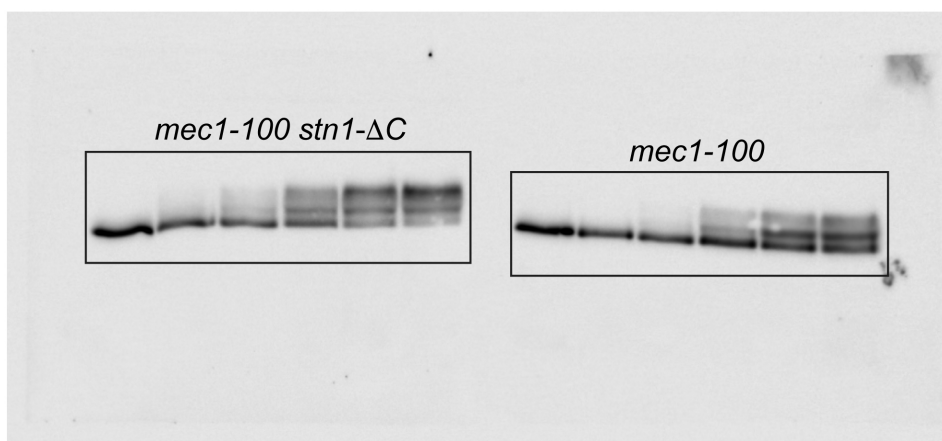

**Figure 9B**

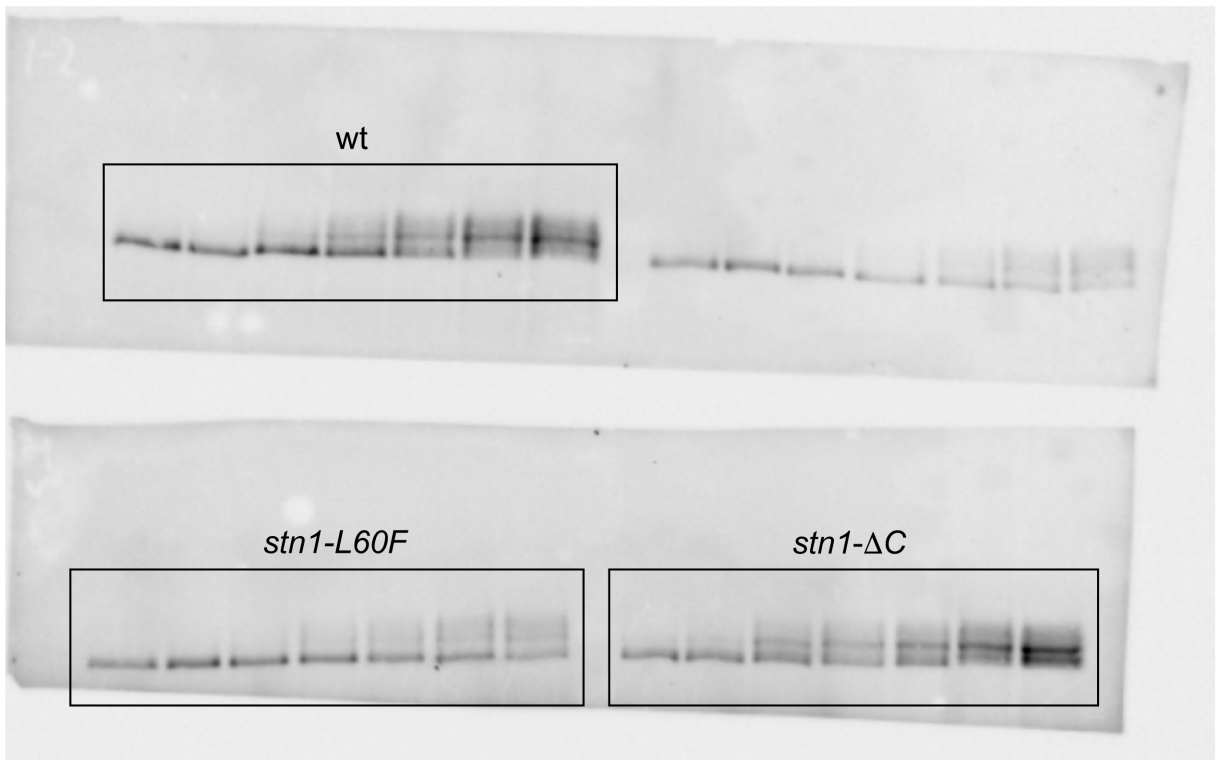

Supplement: S2 Data — (PDF) [file pgen.1011917.s010.pdf]
