# Supplementary material for: Stn1 supports Mec1 function in protecting stalled replication forks from degradation
Source: PLoS Genet. 2025 Oct 15;21(10):e1011917. doi: 10.1371/journal.pgen.1011917 (PMC12548912; doi:10.1371/journal.pgen.1011917)
Supplement: Attachment — Submitted filename: Response to reviewers.docx [file pgen.1011917.s012.docx]

**Response to reviewers’ comments**

We thank the reviewers for their thoughtful and constructive feedback. We have revised the manuscript accordingly and respond to each point below.

**Reviewer #1:**

*This is an excellent study of one feature of replication fork biology in budding yeast. The authors design strategies to identify proteins important in fork stability, identified because such proteins restore cell viability in cells with low levels of dNTP (conferring hydroxyurea (HU) sensitivity) in a mutant-sensitized cell (mec1-100 mutation). In brief, they identify a role for the protein Stn1protein that is a part of a heterotrimeric complex called CST (Cdc13, Stn1, Ten1) that is known to act at telomeres, and now at internals sites as well. CST that binds ssDNA at telomeres to facilitate telomere biology. They identify suppressors of mec1-100 HUs, one of which is Stn1-L60F. They characterize Stn1-L60F, along with a Stn1ΔC mutation, and mutations in other proteins known to have roles in DNA replication fork biology (exo1, sgs1, mre11-125, pol2-216). The data involve growth assays, assays of ssDNA by a qPCR strategy, ChIP of proteins to stalled forks (from a specific origin, ARS607), and at a DSB (HO induced), and westerns of Rad53 checkpoint protein kinase. The data are impeccable, as per usual from the Longhese lab.*

*Comments:
1. I did have a hard time tracking a certain part of their narrative: The paper would benefit from a model!!! I think a model might be a challenge to draw up for one feature of their data that confused me. Generally, Stu1-L60F binds ssDNA, prevents Sgs1, Exo1 and Mre11 from binding, and therefore prevents these nuclease/nuclease-associated proteins from converting dsDNA to ssDNA. All this in the context of the mec1-100 hypomorphic mutation. Fair enough.*
We have added a working model (new **Fig. 10**).

*What I did not understand is the data in Figure 2, 3 and Fig4. First off, they don't show ssDNA data for mec1-100 sgs1, mec1-100 exo1, mecI-100 mre11-H125N (I think they didn't--they should explain why they don't). So I infer that formation of ssDNA requires these three proteins.
Then, my confusion. Just looking at Figure 4 and Fig 6 for Stn1L60F: In a mec1-100 mutant, Stn1L60F prevents formation of ssDNA and increases HU viability. But they wisely add to that double mutant a mutation in sgs1 or exo1 pr mre11-H125N (e.g. triple mutant mec1-100, Stn1L60F exo1), HU viability is low! Thus, the HU viability in mec1-100 Stn1 L60F requires intact Sgs1, Exo1, and Mre11 (as mre11-125)! Yet the model is that Stn1-L60F prevents/minimizes Sgs1, Exo1, Mre11 from binding ssDNA near stalled forks? The authors should provide a plausible explanation for this data, and provide a model!!!*

To avoid overcrowding in the main figures, we initially omitted to show ssDNA in *mec1-100 sgs1, mec1-100 exo1* and *mec1-100 mre11-H125N*. We now provide ssDNA measurements in S2 Fig, showing that *exo1Δ, sgs1Δ,* and *mre11-H125N* each reduce fork-proximal ssDNA in *mec1-100*, consistent with their roles in generating the ssDNA signal.

Our results support a two-tier mechanism at HU-stalled forks in *mec1-100*: (i) excessive, fork-proximal nuclease engagement is detrimental (runaway resection/ssDNA accumulation); (ii) a basal processing activity remains beneficial for remodeling/restart. Stn1-L60F does not abolish nuclease function; it shields fork-proximal DNA to prevent hyper-resection while permitting basal activity. Consequently, removing Exo1, Sgs1, or Mre11 eliminates both harmful and beneficial components—worsening *mec1-100* survival and abrogating Stn1-L60F-mediated suppression. We now discuss this point (lines 213-222 and 358–369).

*And, what about Stn1 in the CST complex (with Cdc13 and Ten1). They should at least speculate on if they think Stn1 is acting with Cdc13 and Ten1 at stalled forks, or is acting alone, or is unknown.*

We now provide AlphaFold3-guided modeling with HADDOCK refinement (new Figure 6C, D and E). L60F maps to the Stn1 N-terminal OB fold and increases predicted contacts with ssDNA; modeling also suggests interface adjustments that enhance Cdc13–DNA interactions within CST. These observations support a model in which Stn1 is acting within the CST complex and not alone. We have discussed this point (lines 397-402).

*The paper for this reviewer is otherwise solid and extremely well done.*

**Reviewer #2:**

*This is a fascinating paper, adding much to our understanding of the regulation of end-resection at telomeres, at stalled replication forks and at double-strand breaks. The role Stn1 (and presumably the rest of CST) is an unexpected complication in our understanding of end-processing in yeast. Apparently in contrast to recent conclusions concerning mammalian CST, where CST is viewed as filling-in the just-resected end, in yeast it appears that CST more directly impairs all of the several exonuclease activities. An apparently important difference that emerges from this study is how Mre11, Exo1 and Sgs1 affect the formation of ssDNA in replication-arrested cells versus the creation of a double-strand break. In end-processing of a DSB, Sgs1 (with Dna2) and Exo1 are usually viewed as redundant and only in a double mutant is there a severe effect on long-range resection. The results presented here imply that at stalled replication forks, both Exo1 and Sgs1 are required, in addition to Mre11, as mutants of each mask both the effects of Stn1-L60F and Stn1-ΔC. This is surprising and confusing; the authors might provide more discussion of this point.*

We now emphasize that, unlike long-range DSB resection, processing at HU-stalled forks in *mec1-100* appears to require contributions from Mre11, Exo1, and Sgs1–Dna2 (lines 370-379). We propose that this reflects the distinct DNA structures at reversed/remodeled forks.

*Specific comment*

*1) If Stn1 acts apparently upstream of all three exonucleases, how is it recruited to ssDNA/DSB regions? At telomeres, Yku70/80 is a prominent resident and it is recruited to DSBs quickly. Does Stn1 recruitment depend on Yku70/80?*

Our results indicate that Stn1 recruitment does not depend on Ku70/80. ChIP in ***ku70*Δ** cells showed unchanged Stn1 binding (new Figure 6A). In addition, our AlphaFold3 analysis of Stn1–DNA interactions (new Figure 6C, D and E) indicates that L60F strengthens the predicted contact with ssDNA, supporting direct recruitment of Stn1 to ssDNA.

*2) Fig. 2. We don’t understand the plots of enrichment of ssDNA at ARSs. Shouldn’t these values be normalized to 1 at t = 0, as in the plots above in the same figure?*

In Fig. 2A, ssDNA at ARSs is normalized to the initial time point (t=0). In contrast, Fig. 2B (now Fig. 6A) reports Stn1 ChIP enrichment at ARS305/ARS607 as relative fold-enrichment, which is not normalized to 1 at t=0.

*3) Another key point in the MS is that the effects of Stn1 mutants are independent of activating the DNA damage checkpoint, even though Rad53 activation is quite strongly affected. In that case, what are the phenotypes of the mutants with mec1 sml1?*

*stn1-L60F* does not suppress HU sensitivity of *mec1Δ sml1Δ* cells (new S1A Fig and lines 153-155). Whether this lack of suppression reflects the much more severe HU sensitivity of *mec1Δ sml1Δ* compared with *mec1-100* or a requirement for basal checkpoint activity is very difficult to assess.

*4) Fig. 8 : the assays for ssDNA are not coincident with the positions of the ChIPs for the exonucleases. Why were different primers used? Are these from the same experiments, and the same side of the DSB?*

Primer choice for ssDNA quantification was constrained by the need for suitable restriction sites for the qPCR-based assay. ChIP primers were originally optimized to detect protein binding close to the HO-induced DSB. Although the primer sets differ, experiments were conducted in parallel on the same side of the DSB. Fig 8 is now Fig 9.

*5) It would be good to show the effect of Stn1 and the nucleases for some DSB repair event, as the effects on resection, though apparently almost all statistically significant, are surprisingly modest, whereas the effects on Rad53 phosphorylation are strikingly large. More generally, how to explain how these small differences have such large effects.*

The decrease in ssDNA generation at the HO-induced DSB is not sufficient to impair SSA-mediated repair. We agree that the reduction in Rad53 phosphorylation in *stn1-L60F* appears more pronounced than the reduction in ssDNA, raising the possibility that Stn1 **can dampen checkpoint activation independently of ssDNA.** However, given that *stn1-L60F* also reduces ssDNA at the DSB, we cannot conclude ssDNA-independent regulation.

*6) cdc13-1 strains arrest but then adapt (Toczyski et al. 1997). Does Stn1-L60F or ΔC affect this behavior? Also, if Stn1-ΔC exhibits the same end-protection defects as ts mutants of cdc13 (l. 308), in what way is Cdc13 more essential to telomere maintenance?*

Our data do not imply a hierarchy of importance among CST subunits. Rather, allele-specific phenotypes (e.g., *cdc13-1* ts vs *stn1-L60F/stn1-ΔC*) perturb distinct CST modules. Cdc13 provides high-affinity telomeric capping, telomerase recruitment, and scaffolding for CST–Polα/primase, whereas Stn1 (with Ten1) primarily limits nuclease access and promotes Polα/primase-dependent fill-in. The severity of certain *cdc13* ts phenotypes reflects the specific functions compromised, not greater essentiality of *CDC13* per se.

Regarding the adaptation, we have analyzed the behaviour of *stn1-ΔC* and *stn1-L60F* mutants. Because checkpoint activation is impaired, the effect of *stn1-L60F* is difficult to evaluate, whereas *stn1-ΔC* cells adapt. However, since the *stn1-L60F* result is not conclusive and the adaptation analysis is not well integrated with the rest of the manuscript, we prefer not to include these data.

*7) Is Stn1-ΔC dominant over stn1-L60F or vice versa? Does this relationship help suggest how many copies of the complex may act at any one site?*

The heteroallelic combination (***stn1-ΔC/stn1-L60F***) exhibits an intermediate phenotype relative to the single mutants. This makes stoichiometric inference difficult and we therefore did not mention this data in the manuscript.

*8) This last point is based on a recent preprint about yeast end-resection and actin:* [*https://www.researchsquare.com/article/rs-5125308/v1*](https://www.researchsquare.com/article/rs-5125308/v1) *Given recent studies of how the Arp2/3 complex blocks resection in yeast, as it apparently does in mammalian cells, one can only wonder how those results are tied into the role of Stn1. The authors might want to comment on this.*

We appreciate the suggestion. As Arp2/3 appears to act as a positive regulator of DSB resection, a thorough discussion would require integrating multiple positive regulators of resection, which is beyond the scope of the present work.

*9) the authors might also comment on the recently published paper:* [*https://www.cell.com/action/showPdf?pii=S2666-979X%2825%2900203-4*](https://www.cell.com/action/showPdf?pii=S2666-979X%2825%2900203-4)

We have now cited and discussed this work in the revised manuscript.

**Reviewer #3:**

*In this study Casari et al, investigated the link between CST complex and checkpoint response in response to replication stress. With a genetic screen they identified a mutation of STN1 (stn1-L60F) that slightly restores viability of mec1-100 cells in response to HU. They showed that Stn1 limits the accumulation of ssDNA at replication forks, counteracting resection by Mre11, Exo1 and Sgs1/Dna2.*

*It would have been fair to state in the introduction that human CST complex was already known to protect stalled replication forks by directly blocking MRE11 degradation of nascent-strand DNA. On the top of that, several studies reported also that Stn1-Ten1 protect blocked forks by antagonising Exo1-extensive resection at DNA ends in fission yeast. Knowing that, the novelty of this study is less. This work rather brings some insight on the connection between Mec1-depedent checkpoint response and Stn1 which is interesting although not fully addressed. However, I have the impression that only the surface has been touched and that we reinvent the wheel in several aspects. The authors claimed that association of Stn1-L60F is increased at the fork however the demonstration by ChIP doesn’t look very convincing. I am not convinced either by the fact that role of protection by Stn1 is independent on the fill-in reaction. Along the same line, this work is also in direct contradiction with a recent study that shows that CST acts after resection at DSB (DOI: 10.1016/j.xgen.2025.100947).*

*Overall, although the discovery of this new allele of stn1 seems interesting, the novelty of the proposed mechanism is questioned and it is not clearly established how this mutation impacts the function of CST.*

The point that human CST protects stalled replication forks by blocking MRE11-mediated degradation of nascent DNA is already stated in the Introduction (see lines 102–103 of the original submission). The studies on *S. pombe* Stn1–Ten1 antagonizing Exo1-mediated extensive resection at telomeres have been mentioned in the revised version (references 53,55).

Regarding fill-in synthesis, because we observe an increase in ssDNA at stalled forks in *pol12-216* (Fig 6 in the original manuscript) and in the double mutant *pol12-216 pol1-236* (Fig 7 of the revised version), we have revised our interpretation to reconcile and complement our work with the recently published study showing that Stn1 limits ssDNA at a Cas9-induced DSB through its interaction with Polα–primase (DOI: 10.1016/j.xgen.2025.100947).

Finally, we added an AlphaFold3 analysis of the L60F mutation effect on DNA binding (Fig 6C, D and E), supporting the experimentally observed increase in Stn1-L60F association at both stalled replication forks and DNA DSBs.

*Specific points:*

*In figure 1, it will helpful to insert a scheme of the CST elements with functional domains and their own domain of interaction and interaction with polymerase alpha (Pol1 and Pol12), the telomerase, DNA,… L60 should lie in the Ten1 interaction domain. Alphafold modelling is recommended for this kind of analysis. How does this mutation impact interaction with Ten1 and Cdc13 and ssDNA binding? This was not assessed and discussed.*
We have included a scheme of CST domains (Figure 6B) and an AlphaFold 3 structural prediction (Figure 6C, D and E and S1 Table). L60F lies in the N-terminal OB fold, DNA binding domain of Stn1, which is also involved in protein-protein interaction with Cdc13 (Figure 6C). In detail, molecular dynamics-based protocols for molecule relaxation lead to the insight that the L60F substitution in Stn1 not only slightly modifies the contact interface towards Cdc13, but also increases the polar interactions with DNA in both Stn1 and Cdc13 DNA-binding domains, improving the affinity for single-stranded DNA. This observation is consistent with the enhanced binding signal detected in our experiments and provides a structural rationale for the observed phenotype.

*Figure 1A: The rescue of mec1-100 allele by stn1-L60F is mild. Survival curves with statistical analysis will be more appropriate to visualize this rescue and to convince the readers.*

Survival curves have been added (Figure 1B).

*Figure 1E: I don’t see a fundamental change in mec1-100 vs mec1-100 stn1-L60F double. I would say epistatic unless quantifications of Western blot are shown. Thus, the statement that stn1-L60F impairs CP activation is not clearly demonstrated.*

Quantification of Rad53 phosphorylation has been added (Figures 1F and 1H).

*Figure 2B: The demonstration that stn1-L60F reduces ssDNA accumulation seems convincing, but the demonstration that Stn1-L60F accumulates at stalled forks is not. According to Figure 1F, the effect of Phleomycin on the delay of CP activation is stronger. Using Phleomycin could a way to exacerbate the ChIP signal.*

The addition of phleomycin did not improve resolution of the observed differences. Although the binding differences between WT and L60F remain statistically significant and reproducible across independent replicates, we have optimized the ChIP conditions (wash buffers) to better preserve weaker DNA–protein interactions; under these conditions the differences are more evident (see new Figure 6A). The molecular rationale for increased DNA affinity was investigated by AlphaFold3/HADDOCK analyses (new Figure 6C–E; S1 Table).

*Lane 219 p 9 : should be figure 4 ?*

The reference to Figure 3 is correct.

*This statement p9 “Based on these findings, Stn1 may limit ssDNA generation at stalled replication forks by counteracting the resection activity of Mre11, Exo1, and Dna2-Sgs1 via polymerase α–primase-dependent fill-in synthesis and/or by directly inhibiting these nucleases ». The authors need to mention that CST is known to protect stalled replication forks by directly blocking MRE11 degradation of nascent-strand DNA in human cell lines and that reported that Stn1-Ten1 protect blocked forks by antagonising Exo1-extensive resection in fission yeast. This assumption has already been proposed and demonstrated by previous studies.*

We revised the paragraph to clearly acknowledge all prior studies (lines 271-278).

*Figure 6 : I have a concern regarding the interpretation of the data with the pol12-216 allele. The authors states page 9 line 240 that this mutation G325D in Pol12 disrupts the interaction with Polymerase alpha and CST. According to Grossi et al., disruption is only partial (two-fold). In addition, it is likely that Cdc13 still interacts with Pol1 subunit pol12-216 mutant. Why the author did not combine with both pol12-216 with pol1-236 that abolishes interaction between Cdc13 and Pol1. Thus, although the interaction is reduced pol12-216 mutant, fill-in reaction can still occur.*

We tested the ***pol12-216 pol1-236*** double mutant and obtained results consistent with ***pol12-216*** alone (see new Figure 7) that weakening CST–Polα contacts increases ssDNA at HU-stalled forks. However, the L60F mutation still decreased ssDNA in ***pol12-216 pol1-236,* indicating that** the suppression conferred by *stn1-L60F* does not require CST-Polα interface and is likely mediated primarily by an additional mechanism.

*Is possible that L60F mutation compensate the interaction with Pol12-G325D? This was not addressed. The combination of mec1-100 with pol12-216 mutant is synthetic lethal but we can’t rule out that this effect is not due only to a decrease in interaction with CST.*

The ***pol12-216*** allele increases ssDNA in ***mec1-100*** cells, consistent with a contribution of fill-in synthesis to limiting ssDNA. Compensation of *pol12-216* by L60F is unlikely as Stn1 interacts with Pol12 through its C-terminal region, whereas L60F resides in the N-terminal OB fold. In any case, *stn1-L60F* reduces ssDNA also in *pol12-216 pol1-236* cells.

*Figure 8: A recent publication demonstrates the role of CST in fill-in reaction at Cas9-induced DSB (PMID: 40675158). This work clearly overlaps with and contradicts the result of Figure 8. While the authors claim that CST protects from resection independently of fill-in synthesis this new study demonstrates that CST acts after resection to limit ssDNA accumulation by fill-in reaction.*

We do not view the results as contradictory but complementary. The cited study demonstrates that Polα-primase–dependent fill-in limits ssDNA at a Cas9-induced DSB. Our data agree that fill-in contributes: weakening CST–Polα contacts (*pol12-216* in the original submission, and *pol12-216 pol1-236* in the revision) increases ssDNA at stalled forks. At the same time, we find that *stn1-L60F* still suppresses HU sensitivity and reduces ssDNA at stalled forks in *mec1-100 pol12-216 pol1-236*, and decreases Mre11/Exo1/Sgs1 association at forks and at an HO-induced DSB, while showing higher Stn1 occupancy. AF3/HADDOCK modelling supports that L60F enhances ssDNA contacts, consistent with a recruitment-limiting (“shielding”) mechanism that restricts nuclease access. In the revision we therefore present a dual model: CST limits ssDNA both by promoting Polα-primase–mediated fill-in and by restricting nuclease recruitment/retention, with the latter predominating in the *stn1-L60F* allele. This framework reconciles our fork/HO-DSB data with the Cas9-DSB findings in PMID: 40675158.

*Deletion of rif1 is known to restore viability of dysfunctional stn1 alleles by likely firing new origins. How deletion of rif1 would impact mec1-100 and stn1-deltaC alleles. Would it be epistatic or additive when combined to stn1-L60F?*

*rif1Δ* is lethal in *stn1-ΔC* cells (Anbalagan et al. 2011), complicating the analysis. Although analyzing *rif1Δ* with *mec1-100* and *stn1-L60F* would be interesting, a full interpretation would require several additional experiments including testing origin firing and is beyond the scope of the current study.
